# Evolutionary Expansion and Diversification of the *GDSL* Gene Family in Grasses

**DOI:** 10.3390/biology15131005

**Published:** 2026-06-25

**Authors:** Qian Zhang, Xin Wen, Huan Li, Jingjing Zou, Jie Yang, Xuan Cai, Xusheng Gong, Yingting Zhang, Zeqing Li, Hongxi Chen, Li Shi, Yuanhang Wu, Lijun Gong, Haiyan Ma, Hongguo Chen, Xiangling Zeng

**Affiliations:** 1 Key Laboratory of National Forestry and Grassland Administration on *Osmanthus fragrans*, School of Nuclear Technology and Chemistry & Biology, Hubei University of Science and Technology, Xianning 437100, China; qianzhang@hbust.edu.cn (Q.Z.); 19863349824@163.com (X.W.);; 2 Xianning Forestry Academy of Sciences, Xianning 437100, China; 3 Public Inspection and Testing Center, Xianning 437100, China; 4 Land Reserve Center of Gaomi Bureau of Natural Resources and Planning, Gaomi 261500, China

**Keywords:** GDSL esterase/lipase, grass species, comparative genomics, evolutionary analysis, cold stress response, gene duplication, synteny, cis-regulatory element

## Abstract

The glycine-aspartic acid-serine-leucine (GDSL) esterase/lipase family is a functionally diverse group of hydrolytic enzymes involved in multiple plant biological processes, including stress adaptation and development. However, its evolutionary patterns, functional conservation, and stress-responsive mechanisms in grasses remain not fully elucidated. In this study, a comprehensive comparative genomic analysis was performed on the *GDSL* gene family across nine representative grass species and *Arabidopsis thaliana*. Genome-wide identification, phylogenetic analysis, duplication pattern detection, synteny analysis, cis-regulatory element prediction, protein–protein interaction (PPI) network construction, and RNA-seq-based expression profiling were employed. A total of 1707 *GDSL* genes were identified, with substantial expansion in grasses, especially hexaploid wheat. Whole-genome and segmental duplications were the major drivers of family expansion, with most duplicated genes under strong purifying selection. A grass-specific clade (C3-2) was identified, and extensive syntenic conservation was observed among closely related grasses. Promoter analysis revealed enrichment of stress- and hormone-responsive cis-elements, and RNA-seq showed dynamic GDSL expression under low-temperature stress in rice and wheat. These findings demonstrate that the expansion of the *GDSL* gene family in grasses is driven by polyploidization and lineage-specific duplication, accompanied by the emergence of a grass-specific clade (C3-2) and regulatory diversification, collectively shaping stress-responsive evolutionary innovation in Poaceae.

## Simple Summary

We explored a key class of functional genes across nine typical grass crops and the model plant Arabidopsis. We identified 1707 members of this gene group and found its obvious gene number growth in grasses, especially in polyploid wheat. Gene replication events mainly drove the expansion of this gene family, and most retained genes remained stable and conservative during evolution. Closely related grass species share highly similar gene distribution, and some gene subgroups uniquely formed in grasses. These genes carry abundant regulatory signals related to stress and hormone response, and many members show obvious expression changes under low temperature in rice and wheat. Our findings clarify the evolutionary characteristics and stress response roles of these genes in grasses, and provide a useful gene resource for cultivating stress-tolerant cereal crops in future agricultural breeding.

## 1. Introduction

Glycine-aspartic acid-serine-leucine (*GDSL*) proteins constitute a distinct subfamily within the serine-glycine-asparagine-histidine (*SGNH*) hydrolase superfamily, defined by a highly conserved Gly–Asp–Ser–Leu (GDSL) motif near the N-terminus [1,2,3]. This structural feature distinguishes them from classical lipases harboring the glycine-any amino acid-serine-any amino acid-glycine (*GXSXG*) motif, endowing *GDSL* with exceptional catalytic flexibility and broad substrate specificity to hydrolyze diverse lipid-derived and ester-containing compounds [1,4]. Owing to these versatile catalytic properties, *GDSL*s are evolutionarily conserved across bacteria, fungi, animals, and plants, participating in core biological and metabolic processes across kingdoms [1,3,5]. Recent studies have expanded the acyltransferase landscape in plants, confirming that *GDSL* enzymes cooperate with benzyl alcohol O-acetyltransferase/anthocyanin O-hydroxycinnamoyltransferase/hydroxycinnamoyl-CoA shikimate/quinate hydroxycinnamoyltransferase/dihydroflavonol 4-O-reductase (*BAHD*) acyltransferase and serine carboxypeptidase-like (*SCPL*) families to maintain lipid and metabolite homeostasis [6,7,8,9].

In plants, GDSLs have emerged as key regulators of fundamental physiological and developmental programs. Recent functional studies have validated their critical roles in seed germination, pollen development, cuticle formation, cell wall remodeling, secondary metabolism, and lipid homeostasis [10,11,12]. For instance, the rice *BS1* (*brittle leaf sheath1*) gene encoding a *GDSL* modulates cell wall biosynthesis and mechanical strength [13], while Arabidopsis *GDSL*s govern cutin biosynthesis, epidermal patterning, and reproductive development [10,11]. Beyond development, *GDSL*s are central to plant stress resilience: their expression is strongly induced by drought, salinity, cold, pathogen invasion, and wounding [14,15,16,17]. Overexpression of specific *GDSL* genes enhances abiotic stress tolerance by regulating cuticular wax deposition, membrane lipid stability, and reactive oxygen species (ROS) scavenging [14,16], while other family members directly bolster plant immunity by activating systemic resistance and antimicrobial defenses [18,19,20,21]. Natural variation in *TaGDSL-7D* in wheat regulates grain weight and yield, and *ZmSWL3* in maize regulates drought resistance through endoplasmic reticulum localization [21,22]. *GDSL* genes also improve saline–alkali resistance by enhancing lignin synthesis and ROS balance [8]. Collectively, these studies demonstrate that plant *GDSL*s have undergone extensive functional diversification, acting as critical molecular links between development, metabolism, and environmental adaptation [12,13].

The evolutionary expansion and functional divergence of the *GDSL* gene family are tightly coupled to plant genome evolution. With the proliferation of high-quality plant genome assemblies over the past five years, genome-wide analyses have confirmed that *GDSL* genes form large multigene families across angiosperms [12,23,24]. Marked variation in *GDSL* copy number across species indicates that lineage-specific expansion has shaped the family’s evolutionary trajectory [16,25]. Tandem duplication, segmental duplication, and whole-genome duplication (WGD) have collectively driven *GDSL* expansion in soybean, cotton, Rosaceae species, and other lineages [16,23,25]. Notably, duplicated *GDSL* paralogs frequently diverge in exon–intron architecture, conserved motif composition, and spatiotemporal expression, indicative of widespread subfunctionalization and neofunctionalization during evolution [16,23,24]. Cross-species annotation reveals that the *GDSL* hydrolase family has conserved motifs and low sequence similarity in plants [26]. *GbGDSL* in cotton and *GDSL53* in Arabidopsis further confirm the functional differentiation of family members in cell elongation and polysaccharide acetylation modification [27,28].

Grasses (Poaceae) represent one of the most evolutionarily successful and economically vital plant families, encompassing staple cereal crops that sustain global food security [29,30]. Grass genomes have undergone recurrent polyploidization, chromosomal rearrangements, transposable element proliferation, and lineage-specific duplication events over the past decade, fostering extensive genomic diversity and functional innovation [29,31,32]. Triticeae species—including bread wheat and its diploid progenitors—experienced multiple rounds of polyploidization, a major driver of gene family expansion and adaptive evolution [29,32,33]. Comparative genomic analyses further reveal that grass genes derived from WGD or tandem duplication often evolve specialized functions or stress-responsive regulatory patterns [33,34], making grasses an exceptional model for dissecting how gene duplication fuels multigene family diversification. In Poaceae crops, *AmGDSL1* in *Agropyron mongolicum* and haplotype differentiation of the *OsGDSL* family in rice are directly related to environmental adaptive evolution [35,36]. Genetic analysis of peduncle length in sorghum and yield traits in rapeseed also confirm the association between Poaceae gene family expansion and agronomic trait improvement [37,38].

Despite substantial progress in characterizing *GDSL* families in individual plant species since 2021, most research has focused on single-genome analyses or targeted gene functional studies [16,23,25]. Comprehensive comparative evolutionary analyses of *GDSL* genes across representative grasses remain scarce. Critical knowledge gaps persist regarding the phylogenetic relationships, duplication dynamics, selective pressures, and lineage-specific diversification of Poaceae *GDSL* genes. Moreover, the evolutionary conservation and divergence of *GDSL* genes between polyploid Triticeae and diploid grass lineages have not been systematically explored, hindering a unified understanding of their adaptive evolution in cereals.

In this study, we address this gap by systematically investigating the evolutionary mechanisms underlying the expansion and functional diversification of *GDSL* genes in Poaceae including *Aegilops tauschii*, *Hordeum vulgare*, *Triticum aestivum*, *Triticum urartu*, *Oryza sativa*, *Brachypodium distachyon*, *Zea mays*, *Sorghum bicolor*, and *Setaria italica*. Specifically, we focus on polyploidization-driven gene family expansion, the emergence of grass-specific evolutionary lineages, and regulatory divergence associated with environmental stress responses. We selected rice and wheat to explore *GDSL* expression under cold stress due to its agricultural importance and abundant related omics data, while other stresses and species will be studied later. Our analyses reveal that *GDSL* gene evolution in grasses is shaped by coordinated genomic and regulatory processes, providing new insights into the evolutionary innovation of this gene family in monocots.

## 2. Materials and Methods

### 2.1. Genome Data Collection and Identification of GDSL Genes

Genome assemblies and corresponding annotation files of eight representative grass species, including *Brachypodium distachyon* (v3.1), *Oryza sativa* (IRGSP-1.0), *Sorghum bicolor* (v3.1.1), *Setaria italica* (v2.2), *Triticum aestivum* (IWGSC RefSeq v1.1), *Triticum urartu* (v1.0), *Aegilops tauschii* (v1.0), and *Hordeum vulgare* (IBSC v2), were retrieved from Phytozome v13 and Ensembl Plants v54. The *Arabidopsis thaliana* genome (TAIR10) was downloaded from TAIR and used as a dicot outgroup for comparative evolutionary analysis [39,40,41].

For systematic identification of *GDSL* esterase/lipase genes, the hidden Markov model (HMM) profile of the conserved *GDSL* domain (PF00657) was obtained from InterPro [42]. HMMER v3.3.2 was used to identify candidate GDSL proteins using the Hidden Markov Model profile of the GDSL domain (PF00657), with an E-value threshold of 1 × 10^−10^ and a minimum domain coverage of ≥70% [43]. To improve identification sensitivity and reduce false negatives, BLASTP (https://blast.ncbi.nlm.nih.gov/Blast.cgi (accessed on 20 April 2026))searches were additionally performed using all annotated *Arabidopsis thaliana* GDSL proteins as queries, with an E-value cutoff of ≤1 × 10^−10^, sequence identity ≥ 30%, and coverage ≥ 50% [44]. All candidate sequences were further validated using Pfam-scan v1.3 to confirm the presence of the complete GDSL (PF00657) domain. Only proteins passing all filtering steps were retained for downstream analyses. For genes with multiple transcript isoforms, the longest protein isoform was retained using the R package seqfinder for downstream analyses [45]. Protein physicochemical properties (molecular weight, pI, instability index) were calculated using ProtParam (https://web.expasy.org/protparam/ (accessed on 20 April 2026)).

### 2.2. Phylogenetic Analysis of GDSL Proteins

Full-length GDSL protein sequences were aligned using MUSCLE v3.8 with default parameters, and poorly aligned regions were trimmed using TrimAl v1.2 (gap threshold = 0.2) [46]. A maximum-likelihood (ML) phylogenetic tree was constructed using IQ-TREE v2.0.3 with the best-fit model selected by ModelFinder. Branch support was assessed using 1000 ultrafast bootstrap replicates and SH-aLRT tests [47]. The tree was rooted using Arabidopsis GDSL proteins as outgroup and visualized with the R package ggtree v3.2.1. Orthogroup classification was performed using OrthoFinder v2.5.4 with default settings.

### 2.3. Chromosomal Distribution and Gene Duplication Analyses

Chromosomal positions of GDSL genes were extracted from GFF annotations and plotted onto chromosomes using gggenes (https://wilkox.org/gggenes/, (accessed on 21 April 2026)) [48]. Gene density and tandem clusters (distance ≤ 100 kb) were analyzed in each grass genome. Collinearity and duplication events were identified using MCScanX v1.0.0 (https://github.com/wyp1125/MCScanX, (accessed on 21 April 2026)) with BLASTP E-value ≤ 1 × 10^−10^. Inter- and intra-species synteny blocks were visualized using JCVI v1.2.7 and circlize v0.4.18 (https://github.com/jokergoo/circlize, (accessed on 21 April 2026)). Synonymous (Ks) and non-synonymous (Ka) substitution rates and Ka/Ks ratios were calculated using KaKs_Calculator 2.0 with the YN model. Duplicated pairs were classified as tandem, proximal, dispersed, or segmental; Ka/Ks < 1 indicates purifying selection, Ka/Ks = 1 neutral selection, and Ka/Ks > 1 positive selection.

### 2.4. Cis-Regulatory Element Analysis in the Promoters of GDSL Genes

To investigate potential regulatory divergence among GDSL genes, 2-kb genomic sequences upstream of the transcription start sites were extracted as putative promoter regions using custom Python v3.14 scripts. Cis-regulatory elements were predicted using the PlantCARE database [49]. Identified cis-elements associated with phytohormone responsiveness, stress response, light responsiveness, and developmental regulation were statistically summarized and classified into different functional categories. The distribution patterns of cis-elements were visualized using the R v4.5.3 packages ggtree and gggenes.

### 2.5. Protein–Protein Interaction Network Analysis of GDSL Proteins

AraNet2 is a functional gene network framework based on Arabidopsis interaction data and computational inference. It enables orthology-based prediction of conserved functional associations across species. Protein–protein interaction (PPI) networks of GDSL proteins were predicted using the AraNet2 database [50]. Functional annotation of proteins within the PPI network was conducted using EggNOG-mapper [51]. The resulting interaction networks were visualized using the R package ggNetView (https://github.com/Jiawang1209/ggNetView (accessed on 22 April 2026)) [50,51].

### 2.6. Expression Profiling Analysis of GDSL Genes Based on RNA-Seq Data

Publicly available RNA-seq datasets were downloaded from the NCBI Sequence Read Archive (SRA) database (https://www.ncbi.nlm.nih.gov/home/download/ (accessed on 24 April 2026)) under accession numbers PRJNA772921 and PRJNA787922. Raw sequencing reads were filtered and quality-controlled using fastp [52]. Clean reads were subsequently aligned to the corresponding reference genomes using HISAT2 v2.2.1 [53]. Gene expression abundance was quantified using featureCounts and normalized as transcripts per million (TPM) values [54]. Tissue-specific expression patterns of GDSL genes were analyzed across different developmental tissues and conditions. Expression patterns of GDSL genes across different tissues were visualized using the R v4.5.3 package pheatmap.

### 2.7. qRT-PCR Validation of Transcriptome Data

For japonica rice, seedlings were maintained at 17 °C and sampled at 1 d, 2 d, 3 d, and 4 d post cold exposure, alongside untreated control seedlings. For hexaploid wheat, leaf tissues were harvested under a series of gradient cold treatments ranging from 5 °C down to −25 °C (Tn-M5 to Tn-M25). All leaf tissues were immediately frozen in liquid nitrogen after collection and stored at −80 °C. Gene-specific primers targeting unique coding regions of candidate GDSLs were designed via Primer 5.0, with OsActin and TaActin chosen as internal reference genes for rice and wheat, respectively (Supplementary Data S1).

Total RNA was extracted using a commercial plant RNA isolation kit (Vazyme, Nanjing, China), followed by DNase I digestion to eliminate genomic DNA contamination. RNA integrity and purity were checked by agarose gel electrophoresis and NanoDrop spectrophotometry. Qualified RNA was reverse-transcribed into first-strand cDNA using the HiScript II RT kit (Vazyme). The 10 μL qRT-PCR reaction system contained 5 μL 2 × ChamQ SYBR qPCR Master Mix, 2 μL diluted cDNA template, 0.5 μL forward primer, 0.5 μL reverse primer and 2 μL RNase-free water. The amplification program included an initial denaturation step at 95 °C for 3 min, followed by 40 cycles of 95 °C for 10 s and 60 °C for 30 s; melting curve analysis was performed after amplification to confirm single specific amplicons. Each sample contained three biological replicates and three technical replicates.

## 3. Results

### 3.1. Genome-Wide Identification and Physicochemical Features of GDSL Proteins

**Figure 1:**
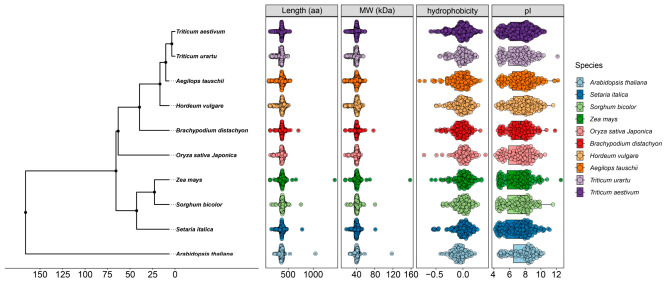
Identification and physicochemical characterization of GDSL family members in 10 species investigated in this study. Protein length, molecular weight, grand average of hydropathicity (GRAVY), and theoretical isoelectric point (pI) were predicted using the ProtParam tool (https://web.expasy.org/protparam/ (accessed on 25 April 2026)).

Genome-wide analysis identified 1707 *GDSL* genes across nine representative grass species and *Arabidopsis thaliana* (Figure 1, Supplementary Data S2), with gene copy numbers ranging from 107 in *A. thaliana* to 522 in hexaploid *Triticum aestivum*. *GDSL* gene copy numbers varied substantially among species, with most diploid genomes containing 107–160 members. The remarkable expansion observed in wheat is likely associated with its allohexaploid origin and extensive gene retention following polyploidization, suggesting that polyploid evolution has played an important role in shaping *GDSL* family expansion in grasses. In contrast, diploid grass species displayed relatively similar *GDSL* gene numbers, indicating a generally conserved family size across Poaceae lineages.

Despite considerable variation in gene copy number, the physicochemical properties of GDSL proteins remained highly conserved among species. Protein lengths ranged from 113 to 1427 amino acids, whereas median lengths were consistently distributed between 367 and 383 amino acids. Similarly, molecular weights varied from approximately 12.8 to 157.8 kDa, while median values remained close to 40 kDa across all species. Hydrophobicity analysis showed that most GDSL proteins were weakly hydrophilic, with median GRAVY values ranging from −0.07 to 0.03. In contrast, theoretical isoelectric points (pI) exhibited broader variation, ranging from approximately 4.1 to 12.6. Notably, grass species showed wider pI distributions than A. thaliana, suggesting greater physicochemical variation in GDSL proteins in grasses. Overall, these findings suggest that expansion of the *GDSL* gene family occurred while maintaining relatively conserved core protein characteristics.

### 3.2. Phylogenetic Classification and Evolutionary Diversification of GDSL Proteins

**Figure 2:**
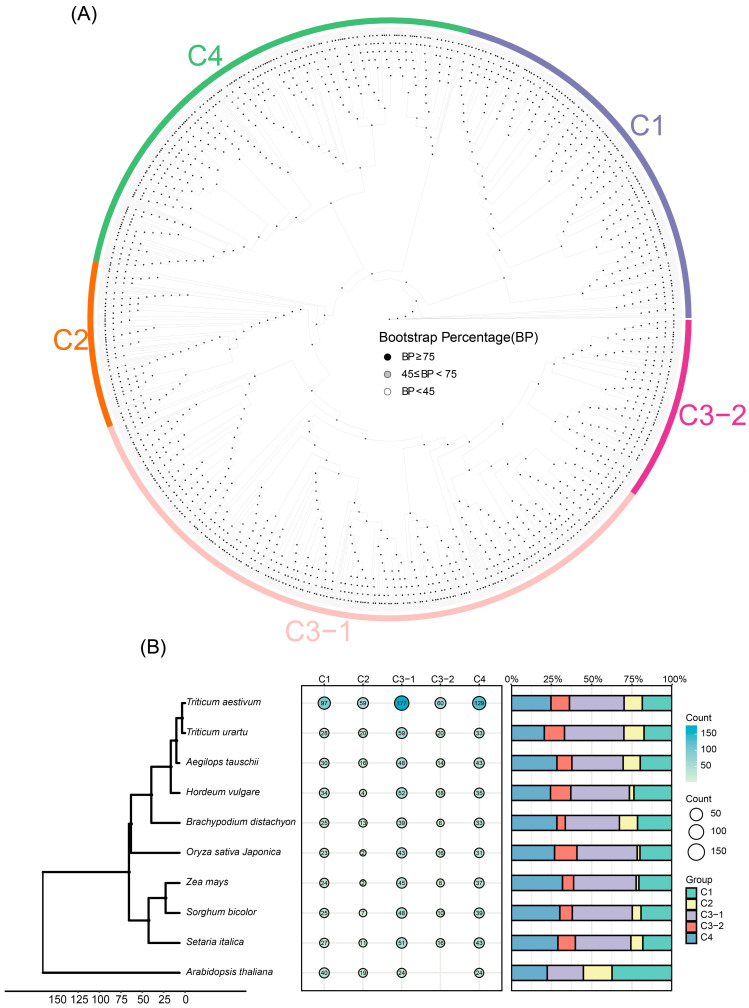
Phylogenetic classification and evolutionary diversification of GDSL proteins in grasses. (**A**) Maximum-likelihood phylogenetic tree constructed using GDSL protein sequences from nine representative Poaceae species and *Arabidopsis thaliana*. (**B**) Comparative distribution of GDSL subgroups among different species.

To investigate the evolutionary relationships of GDSL proteins in grasses, a maximum-likelihood phylogenetic tree was constructed using GDSL protein sequences from nine representative Poaceae species, with *Arabidopsis thaliana* included as an outgroup (Figure 2). Based on phylogenetic topology and branch support, all GDSL proteins were classified into five major clades, designated C1, C2, C3-1, C3-2, and C4. The C3-2 clade was identified as a well-supported sub-lineage within the C3 group based on maximum-likelihood phylogenetic analysis. This clade is strongly supported by both ultrafast bootstrap and SH-aLRT values. All members of this clade are exclusively derived from grass species, and no *Arabidopsis thaliana* sequences are present, indicating a monocot-specific evolutionary lineage within the GDSL gene family.

Substantial differences in gene abundance were observed among the five clades. Among them, C3-1 represented the largest GDSL subgroup and accounted for the highest proportion of members in all analyzed grass species, particularly showing pronounced expansion in *Triticum aestivum*. In contrast, C2 consistently contained the fewest members across species, indicating that this lineage has remained relatively conserved but numerically restricted throughout evolution. Meanwhile, C1 and C4 maintained relatively high and stable gene numbers among species, suggesting that these clades may perform conserved biological functions. Compared with other clades, C3-2 exhibited the greatest variation in copy number among species, especially showing expansion trends in *Triticeae* species, implying lineage-specific gene amplification and retention during grass evolution.

Notably, expansion of the *GDSL* gene family in hexaploid *Triticum aestivum* was highly uneven across clades, with most duplicated genes concentrated in C3-1 and C4. Similar but less pronounced expansion patterns were also observed in *Triticum urartu* and *Aegilops tauschii*. These findings suggest that polyploidization and subsequent gene retention preferentially promoted the expansion of specific *GDSL* subgroups rather than uniformly increasing the size of the entire gene family. Collectively, the asymmetric distribution of GDSL proteins among clades highlights distinct evolutionary trajectories and selective pressures acting on individual *GDSL* lineages during grass evolution.

### 3.3. Chromosomal Distribution and Duplication Patterns

**Figure 3:**
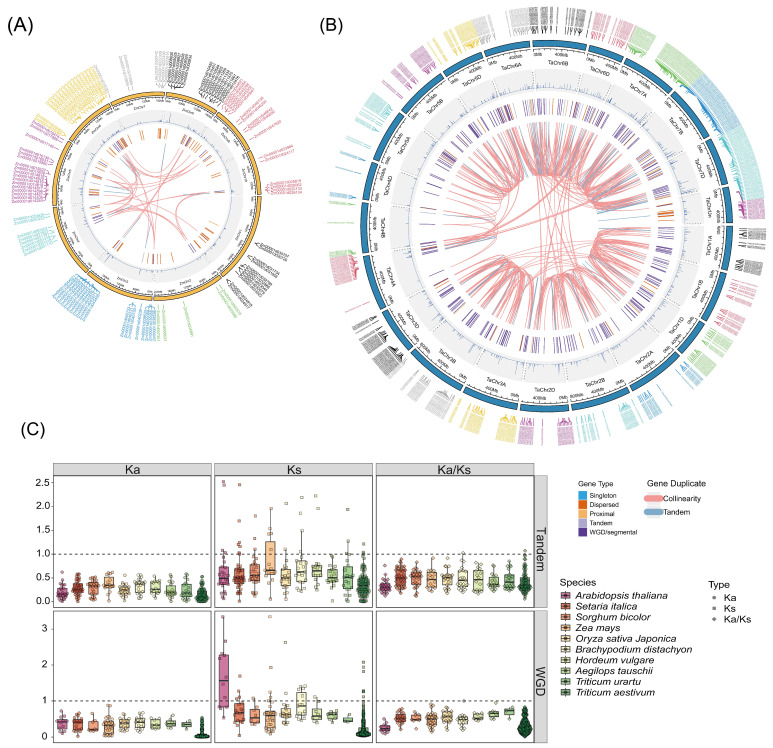
Intra-species collinearity and duplication patterns of *GDSL* genes in grasses. Circos plots showing chromosomal distribution, duplication types, and intra-species collinearity relationships of GDSL genes in representative plant species. (**A**) *Oryza sativa Japonica*; (**B**) *Triticum aestivum*. (**C**) Distribution of Ka, Ks, and Ka/Ks values for tandem duplicated and WGD-derived GDSL gene pairs across different species.

To investigate the evolutionary mechanisms underlying *GDSL* gene family expansion in grasses, gene duplication patterns and intra-species collinearity analyses were performed using MCScanX across the analyzed species (Figure 3A,B, Supplementary Figure S1A–H). Five major duplication categories were identified, including singleton, dispersed, proximal, tandem, and whole-genome duplication/segmental duplication (WGD/segmental). Among these, WGD/segmental duplication represented the predominant expansion mode in nearly all grass species, whereas tandem duplication contributed to local amplification of only a subset of *GDSL* genes (Supplementary Data S3). These results suggest that large-scale genome duplication events have played a central role in shaping the expansion and diversification of the *GDSL* family during grass evolution.

Clear differences in duplication complexity were observed among species with different genome sizes and evolutionary histories. Polyploid wheat (*Triticum aestivum*) exhibited the most extensive collinearity relationships and contained the largest number of duplicated *GDSL* gene pairs, consistent with its recent allohexaploid origin. Similar but less extensive WGD-derived duplication patterns were also detected in *Triticum urartu*, *Aegilops tauschii*, and *Hordeum vulgare*, indicating that recurrent genome duplication and chromosomal rearrangement events contributed substantially to *GDSL* family expansion within *Triticeae* species. In contrast, diploid grasses such as *Oryza sativa*, *Brachypodium distachyon*, and *Setaria italica* displayed comparatively fewer duplicated gene pairs and simpler collinearity structures, suggesting relatively moderate expansion histories.

The duplication types could be clearly distinguished based on gene distribution patterns and gene identifiers. Tandem duplication events were generally characterized by physically adjacent genes with highly similar gene IDs located on the same chromosome, such as TraesCS7A02G535700–TraesCS7A02G535800 and Seita.5G150300–Seita.5G150400, indicating recent local duplication events. In contrast, WGD/segmental duplicated gene pairs were typically distributed on different chromosomes or distant chromosomal regions and often exhibited larger gene ID divergence, such as TraesCS1A02G250600–TraesCS1B02G261200 and TraesCS7A02G388700–TraesCS7B02G290600, reflecting large-scale chromosomal duplication and subgenome conservation following polyploidization. Collectively, these findings indicate that WGD/segmental duplication has been the dominant evolutionary force driving GDSL gene family expansion in grasses, whereas tandem duplication contributed mainly to lineage-specific local diversification.

Ka/Ks analysis further revealed that most duplicated *GDSL* gene pairs exhibited Ka/Ks ratios below 1.0, (Figure 3C) regardless of duplication type, indicating that the majority of duplicated genes have undergone strong purifying selection during evolution. Although a few tandem duplicated gene pairs displayed relatively elevated Ka/Ks values, suggesting possible functional divergence or adaptive evolution, the overall evolutionary pattern remained highly conserved across grass species. These results imply that expansion of the *GDSL* family was accompanied by long-term functional conservation following duplication events.

### 3.4. Comparative Synteny and Evolutionary Conservation of GDSL Genes

**Figure 4:**
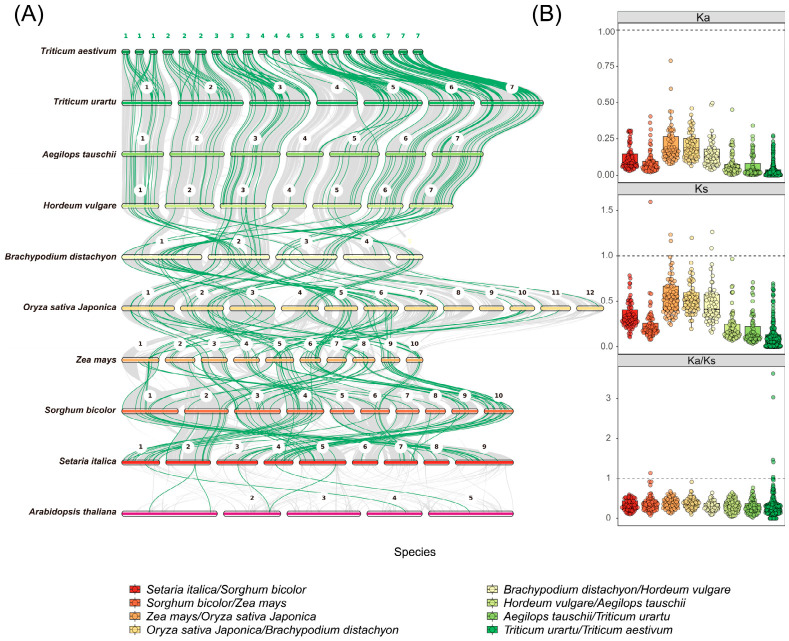
Comparative synteny and evolutionary conservation of *GDSL* genes among representative grass species. (**A**) Cross-species collinearity relationships of *GDSL* genes among representative Poaceae species and *Arabidopsis thaliana*. (**B**) Distribution of Ka, Ks, and Ka/Ks values of orthologous *GDSL* gene pairs among different species combinations.

To further elucidate the conservation and diversification patterns of the GDSL gene family during plant evolution, comparative synteny analyses were performed among different species (Figure 4A). Overall, *GDSL* genes exhibited extensive syntenic conservation among grass species; however, the number of syntenic gene pairs varied markedly across different evolutionary lineages and was closely associated with phylogenetic relationships (Supplementary Data S4). Closely related species generally retained substantially more syntenic *GDSL* gene pairs, whereas only limited collinearity was preserved between distantly related species, reflecting the important roles of genome rearrangement and gene loss in shaping *GDSL* family architecture during long-term evolution.

Among all species combinations, the highest number of syntenic gene pairs was detected between hexaploid wheat *Triticum aestivum* and its closely related ancestral species *Triticum urartu*, with a total of 321 collinear *GDSL* gene pairs identified. This pattern is highly consistent with the evolutionary history of *Triticum urartu* as the A-subgenome donor of wheat, indicating that a large proportion of ancestral GDSL loci were retained during wheat polyploidization. In addition, a relatively high number of syntenic gene pairs was also observed between *Triticum aestivum* and *Hordeum vulgare* (86 pairs), further supporting strong genomic conservation within *Triticeae* species. Among C4 grass crops, *Zea mays* and *Sorghum bicolor* shared 81 syntenic *GDSL* gene pairs, while *Sorghum bicolor* and *Setaria italica* retained 76 collinear pairs, indicating substantial conservation of GDSL loci among closely related grass species. In contrast, syntenic relationships between the dicot species *Arabidopsis thaliana* and grass species were dramatically reduced. For example, only six syntenic *GDSL* gene pairs were detected between *Setaria italica* and *Arabidopsis thaliana*, reflecting extensive genomic restructuring of GDSL loci following long-term divergence between monocots and dicots.

Further Ka/Ks analysis revealed that the majority of syntenic *GDSL* gene pairs exhibited Ka/Ks ratios lower than 1 (Figure 4B), suggesting that these genes have predominantly undergone strong purifying selection during grass evolution. The distributions of Ka and Ks values also differed among species combinations, with closely related species generally displaying lower Ks values, whereas more distantly related species exhibited greater sequence divergence. Collectively, these findings indicate that although the *GDSL* gene family experienced substantial expansion in grasses, its core functions remained highly conserved throughout evolution, while lineage-specific divergence was mainly associated with differential gene retention and sequence evolutionary rates.

### 3.5. Comparative Analysis of Cis-Regulatory Elements in GDSL Gene Promoters

**Figure 5:**
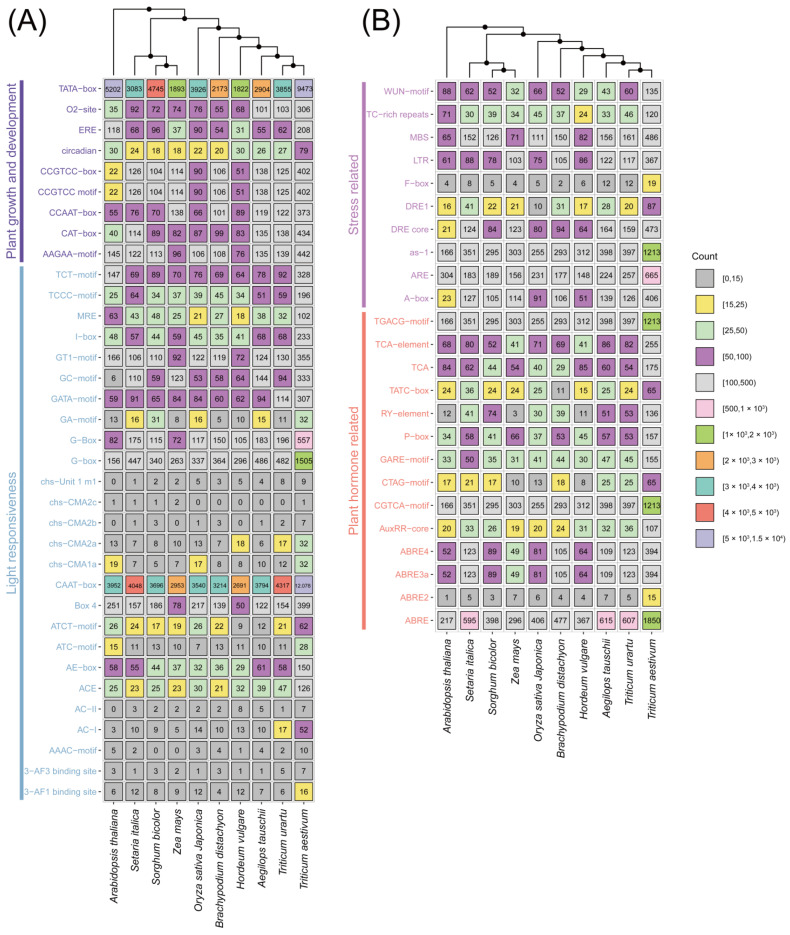
Comparative analysis of cis-regulatory elements in the 2 kb promoter regions of GDSL genes across representative plant species. Cis-elements identified in promoter sequences were classified into four major functional categories, namely, (**A**) light-responsive elements, plant growth and development-related elements; (**B**) plant hormone-responsive elements, and stress-responsive elements.

To investigate the potential regulatory patterns of *GDSL* genes in different plant species, cis-regulatory elements within the promoter regions of *GDSL* genes were systematically analyzed (Figure 5). Based on functional annotation, these elements were mainly classified into four categories, including plant growth and development-related elements, light-responsive elements, plant hormone-responsive elements, and stress-responsive elements. Overall, although the composition of cis-elements was relatively conserved across species, substantial variation in element abundance was observed, which generally correlated with genome complexity and *GDSL* gene family size.

Among all analyzed species, the hexaploid wheat *Triticum aestivum* contained the highest number of cis-regulatory elements, showing remarkable enrichment across nearly all element categories. For example, the numbers of TATA-box and CAAT-box elements reached 9473 and 12,078, respectively, which were substantially higher than those observed in other species. In addition, light-responsive elements such as G-box, GT1-motif, and GATA-motif, together with hormone- and stress-related elements including ABRE, ARE, MBS, and LTR, also exhibited pronounced expansion in wheat. In contrast, *Arabidopsis thaliana* and several diploid grass species, which possess relatively compact genomes and fewer *GDSL* genes, generally showed lower cis-element abundance. Overall, species with larger and more complex genomes, particularly *Triticum aestivum*, *Triticum urartu*, and *Aegilops tauschii*, tended to contain substantially more promoter elements, suggesting that expansion of the *GDSL* gene family was accompanied by large-scale retention and accumulation of regulatory sequences during grass evolution.

From a functional perspective, light-responsive elements represented the most abundant cis-element category across all species, among which G-box, Box 4, and GT1-motif were particularly enriched, indicating that *GDSL* genes may be extensively involved in light-mediated regulatory processes. In addition, hormone-responsive elements such as ABRE, CGTCA-motif, and TGACG-motif were highly enriched in most grass species, implying close associations between *GDSL* genes and phytohormone signaling pathways, especially ABA- and jasmonate-related responses. Among stress-responsive elements, ARE, MBS, and as-1 were broadly abundant in grass species and showed marked expansion in Triticeae lineages, suggesting potential roles of *GDSL* genes in stress adaptation and environmental responsiveness. Collectively, the variation in cis-regulatory element composition among species indicates that expansion of the *GDSL* gene family not only retained core regulatory features but may also have enhanced environmental adaptability through regulatory diversification and promoter evolution.

### 3.6. PPI Network Analysis of GDSL Family Members 

**Figure 6:**
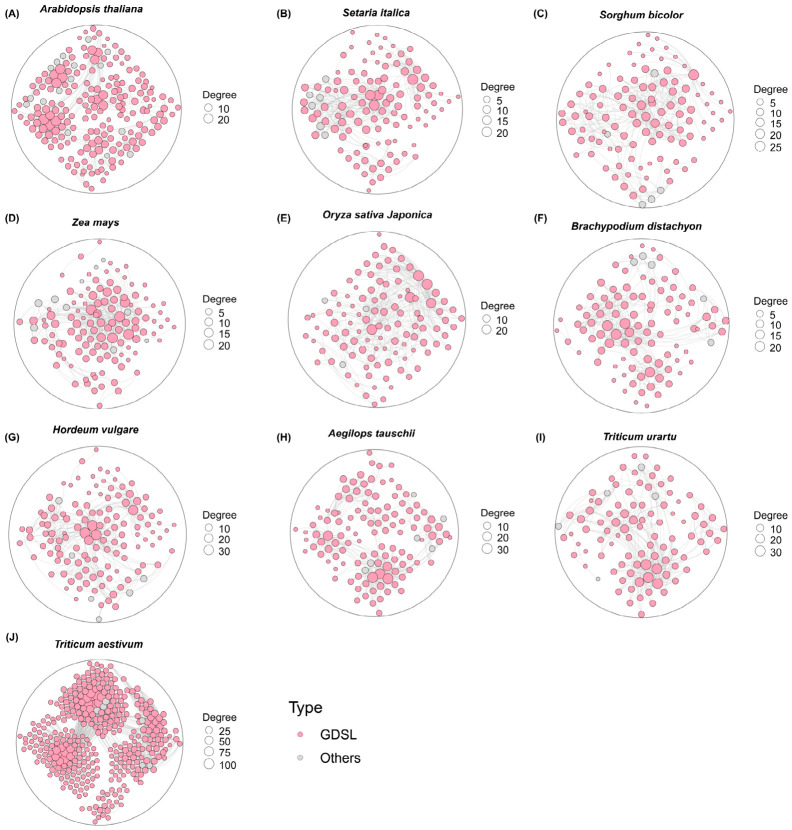
Predicted protein–protein interaction (PPI) networks of GDSL proteins across representative plant species. (**A**) *Arabidopsis thaliana*; (**B**) *Setaria italica*; (**C**) *Sorghum bicolor*; (**D**) *Zea mays*; (**E**) *Oryza sativa Japonica*; (**F**) *Brachypodium distachyon*; (**G**) *Hordeum vulgare*; (**H**) *Aegilops tauschii*; (**I**) *Triticum urartu*; and (**J**) *Triticum aestivum*.

To explore the potential functional associations among GDSL proteins, protein–protein interaction (PPI) networks were constructed for *GDSL* family members in each analyzed species (Figure 6). The PPI networks in this study were predicted via AraNet2, which is built on Arabidopsis interaction data. We used this dicot-based platform because GDSL proteins have evolutionarily conserved structures and core functions in dicots and monocots, and their interacting partners and network patterns are largely conserved across plant species. All predicted networks are orthology-based in silico results and require subsequent experimental verification. Extensive interaction relationships were detected within the *GDSL* family across all species, indicating that GDSL proteins may function through highly interconnected regulatory or metabolic networks rather than acting independently. Most GDSL proteins formed dense interaction clusters with multiple neighboring proteins, suggesting widespread functional coordination among GDSL members during plant growth, development, and environmental responses.

Although the overall interaction architecture was generally conserved, clear differences in network complexity were observed among species. Polyploid and large-genome species, particularly *Triticum aestivum*, exhibited substantially more complex PPI networks, characterized by increased node density and higher interaction connectivity among GDSL proteins. In contrast, diploid species such as *Arabidopsis thaliana*, *Brachypodium distachyon*, and *Oryza sativa* displayed relatively smaller and less interconnected networks. Notably, several highly connected hub proteins were consistently identified in nearly all species, implying that certain GDSL members may play central roles in maintaining conserved biological functions. Collectively, these findings suggest that expansion of the *GDSL* gene family in grasses was accompanied by increased interaction complexity, while the core interaction framework of GDSL proteins remained evolutionarily conserved across plant lineages.

Nodes represent proteins, where pink circles indicate GDSL proteins and grey circles represent predicted interacting proteins. The size of each node represents the degree of the network. Edges indicate predicted functional associations derived from the AraNet2-based orthology inference framework.

### 3.7. Expression Profiling of GDSL Genes Based on RNA-Seq Analysis

**Figure 7:**
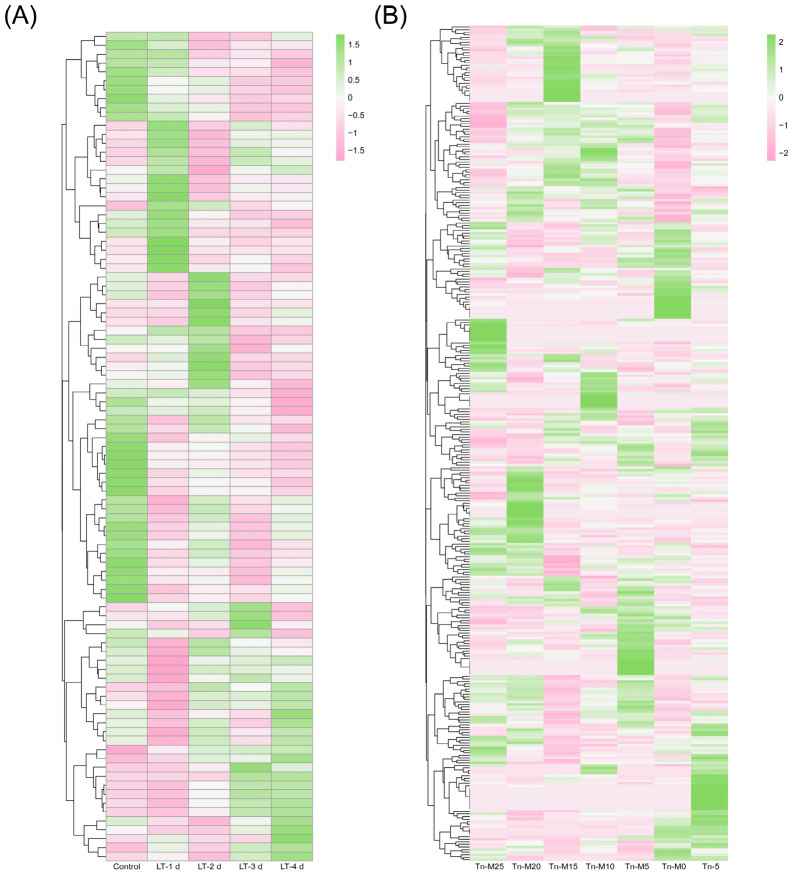
Transcriptional responses of *GDSL* genes under low-temperature stress in rice and wheat. (**A**) Heatmap showing expression profiles of *GDSL* genes in *Oryza sativa* ssp. japonica under low-temperature treatment at different time points, including Control, LT-1 d, LT-2 d, LT-3 d, and LT-4 d. LT indicates low-temperature treatment at 17 °C, and 1 d, 2 d, 3 d, and 4 d represent 1, 2, 3, and 4 days of treatment, respectively. (**B**) Heatmap showing expression patterns of GDSL genes in *Triticum aestivum* under a gradient of low-temperature stress conditions (Tn-M25 to Tn-5), corresponding to 5 °C, 0 °C, −5 °C, −10 °C, −15 °C, −20 °C, and −25 °C, respectively. Expression values were derived from publicly available RNA-seq datasets.

To investigate the potential roles of *GDSL* genes in cold stress responses, publicly available RNA-seq datasets were analyzed in rice and wheat under low-temperature treatments (Figure 7). Expression profiling revealed that a substantial proportion of *GDSL* genes exhibited dynamic transcriptional responses following cold exposure, indicating that GDSL genes show transcriptional responsiveness under low-temperature conditions in grasses. Distinct expression clusters were observed across different treatment stages, with numerous *GDSL* genes showing either rapid induction or strong repression after cold treatment, suggesting transcriptional variation among GDSL members during stress responses.

In rice, multiple *GDSL* genes displayed pronounced transcriptional activation during prolonged low-temperature treatment, particularly at LT-3d and LT-4d stages, whereas several genes were transiently downregulated during the early stress period and subsequently recovered at later stages. Similarly, wheat *GDSL* genes also exhibited extensive expression reprogramming under decreasing temperature conditions, with subsets of genes specifically induced under severe cold treatments (e.g., Tn-M10 to Tn-M25), while others showed preferential expression under mild or recovery conditions. Notably, the transcriptional response patterns in wheat appeared more complex and heterogeneous than those observed in rice, likely reflecting the larger *GDSL* family size and increased regulatory diversification associated with polyploid wheat evolution. Collectively, these findings suggest that *GDSL* genes are transcriptionally responsive to cold stress and may be associated with low-temperature adaptation processes in grasses.

To verify the reliability of the above transcriptome results as proposed by the reviewer, eight rice and eight wheat representative *GDSL* genes with distinct cold-responsive trends were quantified via qRT-PCR (Supplementary Figure S2). All tested genes exhibited consistent up-/downregulation trends between qRT-PCR and RNA-seq data, which confirms the authenticity of the cold-induced transcriptional patterns of *GDSL* family genes.

## 4. Discussion

Plant *GDSL* esterase/lipase family represents one of the most functionally diverse and evolutionarily expanded groups of hydrolytic enzymes, with critical roles in plant development, lipid metabolism, pathogen defense, and abiotic stress adaptation [1,4,12,13]. In the present study, we systematically identified 1707 *GDSL* genes across nine representative grass species and *Arabidopsis thaliana*, revealing substantial and lineage-specific expansion of the *GDSL* family in Poaceae, especially in hexaploid wheat. Previous genome-wide surveys similarly documented remarkable *GDSL* expansion in polyploid crops such as soybean and wheat, supporting the view that recurrent whole-genome duplication (WGD) events are major drivers of the evolutionary diversification of this gene family [16,21]. Our results further demonstrated that despite dramatic copy-number divergence across species, core physicochemical properties—including protein length, molecular weight, and hydrophilicity—remained highly conserved. This evolutionary pattern indicates that functional conservation has been stably maintained during large-scale family expansion, whereas functional diversification likely arose primarily via regulatory evolution and expression divergence rather than drastic structural innovation. Analogous trends have been reported for other large stress-associated gene families in grasses, where polyploidization promoted gene retention while preserving catalytic domains essential for core stress resilience [55].

Phylogenetic reconstruction and duplication analyses further revealed that *GDSL* family expansion in grasses was highly asymmetric among evolutionary clades. Specifically, subgroup C3-1 represented the largest clade in all surveyed grass species, whereas subgroup C2 remained numerically constrained and evolutionarily conserved. More notably, the C3-2 clade was uniquely present in Poaceae and completely absent from Arabidopsis, implying the emergence of grass-specific *GDSL* lineages during monocot evolution, likely associated with adaptive innovations in stress responses and developmental specialization. Similar lineage-specific expansion patterns have been widely observed in grass-adaptive gene families linked to environmental responsiveness and niche adaptation [56]. MCScanX analysis further confirmed that WGD/segmental duplication was the predominant mechanism underlying *GDSL* expansion, especially in Triticeae. This observation aligns closely with the well-established evolutionary history of wheat and related grasses, whose genomes have undergone repeated rounds of polyploidization and chromosomal rearrangement [57]. Moreover, the majority of duplicated *GDSL* gene pairs exhibited Ka/Ks ratios < 1, indicating strong purifying selection has preserved most duplicated copies after expansion. Such evolutionary conservation suggests that most retained *GDSL* duplicates continue to perform essential biological functions, with only a limited subset undergoing neofunctionalization or adaptive divergence to fulfill specialized roles.

The analysis of nonsynonymous (Ka) and synonymous (Ks) substitution rates revealed that most duplicated *GDSL* gene pairs exhibit Ka/Ks < 1, indicating strong purifying selection acting on this gene family in grasses. This pattern is broadly conserved across all analyzed species, suggesting that functional constraints are maintained throughout *GDSL* gene evolution in Poaceae. However, lineage-specific variations were also observed. In particular, a subset of tandemly duplicated genes in polyploid wheat species exhibited relatively elevated Ka/Ks values, suggesting relaxed purifying selection or early-stage functional divergence following recent duplication events. These lineage-specific deviations highlight ongoing subfunctionalization processes in certain evolutionary contexts.

A growing body of evidence highlights the pivotal roles of GDSL proteins in abiotic stress responses, particularly under drought, salinity, and cold conditions [58,59]. Consistent with these prior studies, our promoter cis-element analysis identified substantial enrichment of stress- and hormone-responsive motifs, including ABRE, MBS, LTR, TGACG-motif, and CGTCA-motif, implicating *GDSL* genes in extensive ABA-, jasmonate-, and low-temperature-associated regulatory networks. Notably, Triticeae species displayed pronounced accumulation of these regulatory elements, implying enhanced regulatory complexity during grass evolutionary adaptation. Importantly, several of the identified cis-elements, particularly LTR (low-temperature responsive elements) and dehydration-responsive motifs, are known to be associated with upstream CBF/DREB-mediated cold signaling pathways in plants. This suggests that *GDSL* genes may be transcriptionally regulated by the CBF/DREB regulatory module under cold stress conditions, providing a mechanistic link between promoter architecture and canonical cold-responsive signaling networks in grasses. In parallel, RNA-seq analysis revealed that numerous *GDSL* genes in both rice and wheat exhibited dynamic transcriptional responses to low-temperature treatment, with several genes strongly induced under prolonged or severe cold stress. Wheat exhibited more heterogeneous and complex expression patterns than rice, likely reflecting the larger family size and greater regulatory diversification conferred by polyploid evolution. Previous research has established that cold-induced lipid remodeling and membrane stabilization are pivotal for plant freezing tolerance, and multiple GDSL lipases have been functionally linked to these adaptive processes [16]. Accordingly, the extensive transcriptional reprogramming of *GDSL* genes observed in this study strongly supports our working hypothesis that *GDSL* genes contribute to cold adaptation in grasses through diversified regulatory and metabolic functions. Independent qRT-PCR quantification of 8 rice and 8 wheat genes further verified the reliability of these transcriptomic expression results. Under drought stress, *GDSL* genes in Agropyron mongolicum and maize improve drought resistance by regulating lipid metabolism and stomatal structure. *OsMbl1* in rice reduces blast susceptibility by inhibiting the lipase activity of *OsGdsl1* [60], further verifying the conserved functions of the *GDSL* family in biotic/abiotic stresses.

Future research directions should prioritize three key areas to advance understanding of grass *GDSL* gene function and evolution. First, functional validation of grass-specific C3-2 clade genes via CRISPR/Cas9 mutagenesis and overexpression will clarify their specialized roles in monocot-specific stress adaptation. Second, integrative multi-omics analyses (transcriptomics, lipidomics, and metabolomics) under varying cold stress regimes will dissect the molecular mechanisms by which *GDSL* genes regulate lipid remodeling and membrane stability in temperate grasses. Studies on seed fatty acid reducer genes in soybean and potato tuber development provide methodological references for verifying *GDSL* downstream metabolic pathways [16,61], and lipidome association analysis can expand the substrate and function research of Poaceae *GDSL* [62,63]. Third, population genomic studies across natural grass populations will explore the adaptive significance of *GDSL* gene polymorphisms in cold-prone environments, facilitating the identification of elite alleles for molecular breeding of cold-tolerant cereal crops. Collectively, these efforts will bridge evolutionary patterns and functional mechanisms of *GDSL* genes, providing valuable genetic resources and theoretical foundations for improving abiotic stress resistance in staple grass crops.

## 5. Conclusions

In this study, a comprehensive comparative genomic analysis of the *GDSL* esterase/lipase gene family was performed across nine representative grass species and *Arabidopsis thaliana*. A total of 1707 *GDSL* genes were identified, revealing substantial expansion of the *GDSL* family in grasses, particularly in polyploid wheat. Phylogenetic and duplication analyses demonstrated that whole-genome duplication and segmental duplication were the major evolutionary forces driving *GDSL* family expansion, while most duplicated genes remained under strong purifying selection during grass evolution. Comparative synteny analysis further revealed extensive conservation of *GDSL* loci among closely related grass species, especially within Triticeae lineages. In addition, promoter analysis indicated widespread enrichment of stress-, hormone-, and light-responsive cis-elements, suggesting increasing regulatory complexity accompanying *GDSL* family expansion. PPI networks revealed extensive functional connectivity among GDSL proteins, whereas RNA-seq analyses demonstrated dynamic transcriptional responses of numerous *GDSL* genes under low-temperature stress in both rice and wheat. Collectively, our findings suggest that the evolution of the *GDSL* gene family in grasses is driven by coordinated genomic and regulatory mechanisms. Whole-genome duplication and polyploidization provide the primary source of gene expansion, while lineage-specific retention and regulatory divergence drive functional innovation. The emergence of a grass-specific clade (C3-2) further highlights monocot-specific evolutionary innovation in this gene family.

## Data Availability

The original contributions presented in this study are included in the article; further inquiries can be directed to the corresponding authors.

## Supplementary Materials

The following supporting information can be downloaded at: https://www.mdpi.com/article/10.3390/biology15131005/s1, Figure S1: Intra-species collinearity and duplication patterns of GDSL genes in grasses. Circos plots showing chromosomal distribution, duplication types, and intra-species collinearity relationships of GDSL genes in representative plant species. (A) *Arabidopsis thaliana*; (B) *Setaria italica*; (C) *Sorghum bicolor*; (D) *Zea mays*; (E) *Brachypodium distachyon*; (F) *Hordeum vulgare*; (G) *Aegilops tauschii*; (H) *Triticum urartu*. Figure S2: qRT-PCR quantification validates cold-responsive expression patterns of eight rice and eight wheat GDSL genes under low-temperature stress treatments. Supplementary Data S1: Primer sequences used for qRT-PCR. Supplementary Data S2: Analysis of physicochemical properties of GDSL gene family in 10 species. Supplementary Data S3: Duplicate gene pairs of GDSL gene family in 10 species. Supplementary Data S4: Synteny gene pairs of GDSL gene family in 10 species.
