# Supplementary material for: Evolutionary Expansion and Diversification of the GDSL Gene Family in Grasses
Source: Biology (Basel). 2026 Jun 25;15(13):1005. doi: 10.3390/biology15131005 (PMC13359762; doi:10.3390/biology15131005)
Supplement: Supplementary file 1 [file biology-15-01005-s001.zip › Supplemental Figures.pdf]

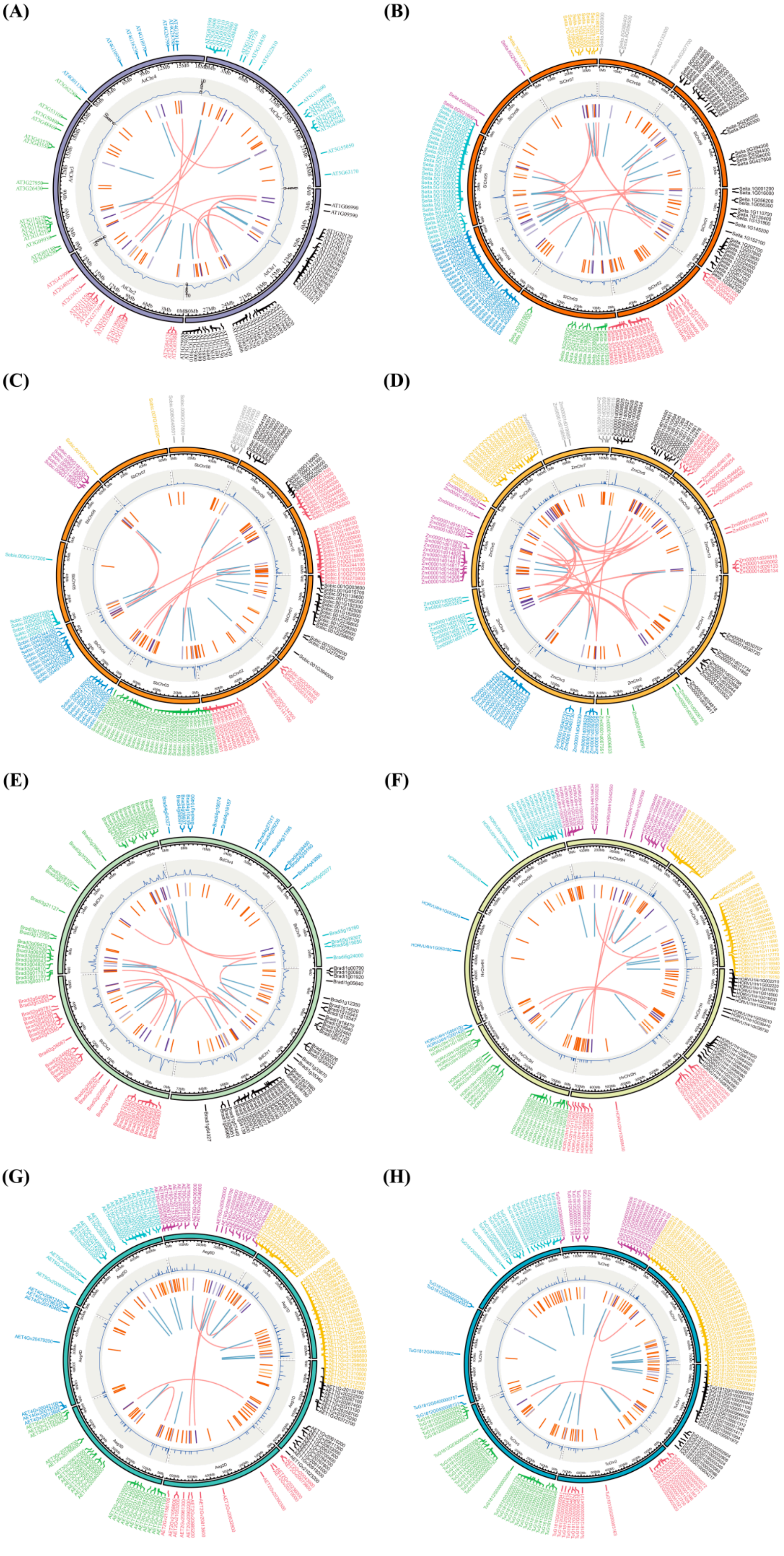
(A)
(B)
(C)
(D)
(E)
(F)
(G)
(H)

Figure S1. Intra-species collinearity and duplication patterns of GDSL genes in grasses. Circos plots showing chromosomal distribution, duplication types, and intra-species collinearity relationships of GDSL genes in representative plant species. (A) *Arabidopsis thaliana*; (B) Setaria italica; (C) *Sorghum bicolor*; (D) *Zea mays*; (E) *Brachypodium distachyon*; (F)*Hordeum vulgare* ; (G)*Aegilops tauschii* ; (H)*Triticum urartu.*

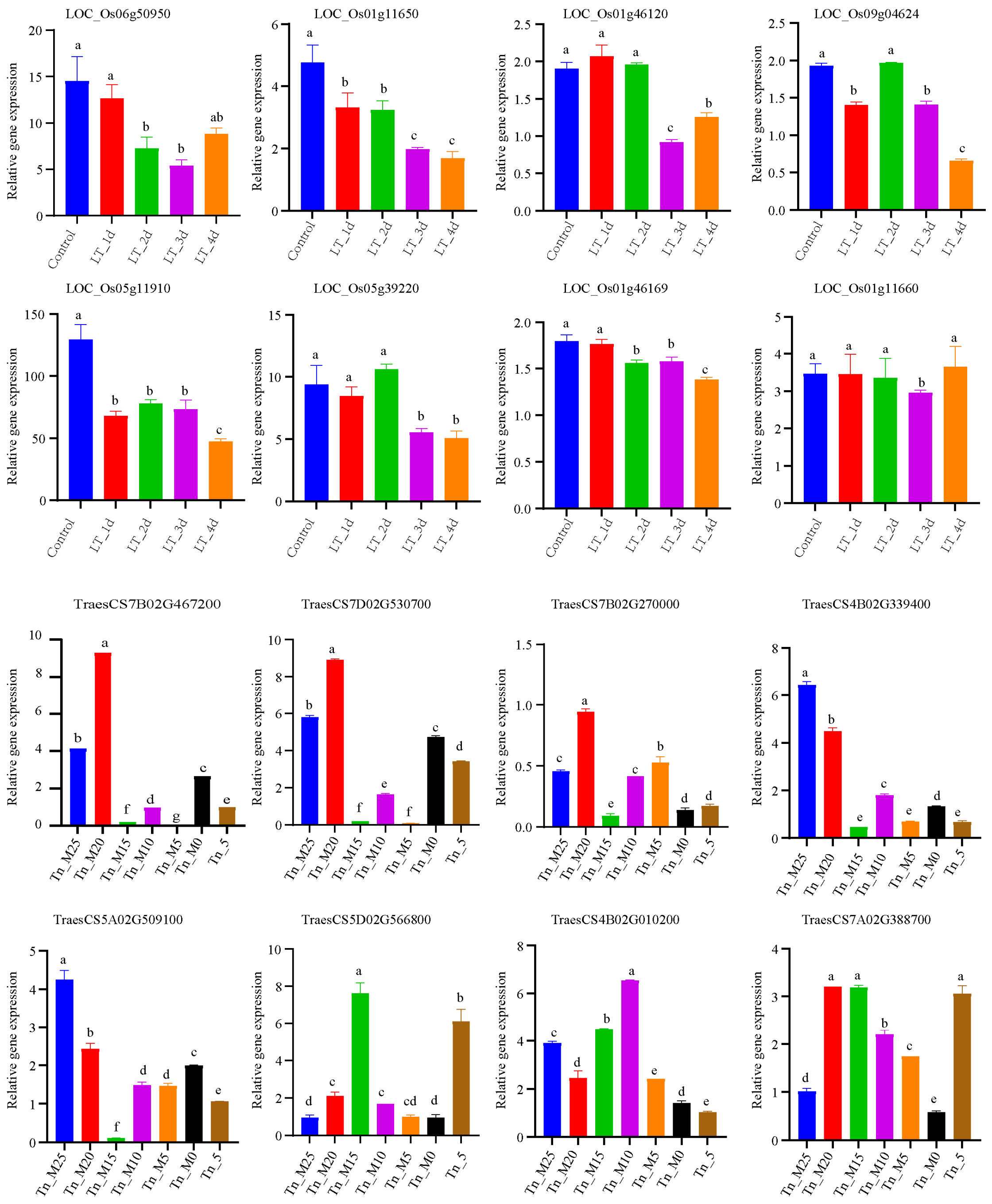

Figure S2. qRT-PCR quantification validates cold-responsive expression patterns of eight rice and eight **wheat GDSL genes under low-temperature stress treatments.** This figure shows qRT-PCR relative expression of eight rice GDSL genes (LOC_Os series, top two rows) and eight wheat GDSL genes (TraesCS series, bottom two rows), performed to verify public RNA-seq data reliability. Rice samples included a normal-temperature control and seedlings treated at 17°C for 1–4 days (LT-1d to LT-4d). Wheat samples covered gradient cold treatments from −25°C (Tn_M25) to 5°C (Tn_M5). Bars show mean values of three biological replicates, and error bars represent standard deviation. One-way ANOVA followed by LSD multiple comparison was used for statistical analysis. Different lowercase letters above bars mark significant differences between treatments ($p < 0.05$). All genes displayed expression trends consistent with transcriptome results, confirming the authenticity of cold-induced transcriptional changes of GDSL family genes in rice and wheat.
